# Dispensing patterns of selective serotonin reuptake inhibitors before, during and after pregnancy: a 16-year population-based cohort study from the Netherlands

**DOI:** 10.1007/s00737-019-0951-5

**Published:** 2019-02-14

**Authors:** Nina Maren Molenaar, Mijke Pietertje Lambregtse-van den Berg, Gouke Jacobus Bonsel

**Affiliations:** 1 The Department of Psychiatry, Erasmus MC, ‘s Gravendijkwal 230, 3015 CE Rotterdam, The Netherlands; 2 The Department of Psychiatry, Icahn School of Medicine at Mount Sinai Hospital, New York, NY USA; 3 The Department of Child and Adolescent Psychiatry/Psychology, Erasmus MC-Sophia, Rotterdam, The Netherlands; 4 The Department of Public Health, Erasmus MC, Rotterdam, The Netherlands; 5 Division Women and Baby, UMC Utrecht, Utrecht, The Netherlands

**Keywords:** Antidepressants, Pregnancy, Dispensing, Serotonin, Pharmacoepidemiology

## Abstract

Management of mental illness in the perinatal period with antidepressants is controversial, since evidence emerged on potential harmful effects to the unborn child. However, over time, the dispensing of antidepressants in the perinatal period has increased. We examined perinatal dispensing patterns over time and the role of a recently issued guideline in this regard. We identified a 16-year cohort of 153,952 Dutch pregnancies with a delivery date between January 1999 and December 2014. Data included exposure to selective serotonin reuptake inhibitors (SSRIs) related to phases of pregnancy (preconception, pregnancy and delivery, post-delivery). The chi-square test for trends was used. With standard logistic regression, we explored the influence of patient characteristics on continuation of SSRIs during pregnancy. A persistent significant rise of dispensing rates in all phases was observed, with the largest increase during pregnancy (from 0.8% in 1999/2000 to 2.1% in 2013/2014, chi-square for trend = 141.735, *p* < 0.001). A substantial change of practice in terms of the SSRI used (less paroxetine) and the policy towards continuation into pregnancy (more continuation over time) was visible. Concomitant use of psycholeptics halved the probability of continuation of SSRIs (OR 0.50, 95%CI 0.43–0.55, *p* < 0.01). Dispensing rates of SSRIs steadily increased last 16 years, especially during pregnancy, caused by an increase in the proportion of women continuing their medication during pregnancy. In view of the demonstrated impact of uncertainty regarding effectiveness and safety of SSRIs in pregnancy, future research should involve more detailed outcome research of SSRIs as it is, and research into viable alternatives.

**Electronic supplementary material:**

The online version of this article (10.1007/s00737-019-0951-5) contains supplementary material, which is available to authorized users.

## Introduction

Management of mental illness in the perinatal period^1^ is an ongoing topic of professional and public debate. Administration of psychotropic medication is a common treatment option (McAllister-Williams et al. 2017), with antidepressants most frequently prescribed (Daw et al. 2012), especially selective serotonin reuptake inhibitors (SSRIs) (Cooper et al. 2007). Over the years, the prescription of perinatal SSRIs increased (Bakker et al. 2008; Charlton et al. 2015; Cooper et al. 2007; Jimenez-Solem et al. 2013), leading to prescription rates of 1.7 to 10.2% during pregnancy (Charlton et al. 2015; Cooper et al. 2007; Hurault-Delarue et al. 2018).

Perinatal SSRI use has become controversial as evidence emerged on potential harmful effects to the unborn child (Simoncelli et al. 2010). Its use has been reported to increased risks for cardiovascular malformations (especially paroxetine) (Grigoriadis et al. 2013b), persistent pulmonary hypertension of the neonate (Kieler et al. 2012), poor neonatal adaptation (Grigoriadis et al. 2013a), preterm delivery, lower birth weight (Ross et al. 2013) and psychiatric disorders in later life (Liu et al. 2017). Untreated depression during pregnancy, however, also bears risk for the child: associations with premature delivery, low birth weight and perinatal mortality have been reported (Grigoriadis et al. 2013c; Grote et al. 2010; Howard et al. 2007). In early childhood, it can lead to behavioural, emotional, cognitive and motor problems (Field 2011; Talge et al. 2007).

Any affected pregnant woman, and their health care professional, therefore faces a complex decision regarding initiation, continuation or discontinuation of SSRIs. Internationally, guidelines agree on a limited set of recommendations, such as the use of psychotherapy as preferred treatment in mild to moderate depression (Molenaar et al. 2018b). But on key aspects of the dilemma, guidelines are unclear (conflicting recommendations or no reference to the topic), i.e., on the first-choice drug and on when and how to switch to another antidepressant, leading to considerable variation in current practice (Molenaar et al. 2018a; Ververs et al. 2009).

In this study, we present population-based information on SSRI dispensing and dispensing patterns related to the pregnancy phases covering 16 years in the Netherlands. We focus on specific SSRIs, expecting a paroxetine decline at the time emerging evidence suggested excess risks of paroxetine on child outcome. Furthermore, we explore the effect of the introduction of the Dutch multidisciplinary guideline in 2012 (NVOG 2012) on continuation of SSRI use during pregnancy. The guideline recommends continuing SSRIs (without switching) in the perinatal period if women are stable on this medication, and psychiatric indication is correct. Lastly, we will examine whether patient characteristics influence the decision to either continue or discontinue SSRIs during pregnancy.

## Methods

### Data sources

For this population-based cohort study, 153,952 Dutch pregnancies were identified after probabilistic linkage between the Outpatient Pharmacy Database of the PHARMO Database Network and the Netherlands Perinatal Registry (PRN). PHARMO is a dynamic cohort of participants that includes drug-dispensing records from community pharmacies of approximately 25% of the Dutch population (Herings et al. 1992). The pharmacies and their populations are generally accepted as representing outpatient drug prescription practice. As part of its national statistical responsibility, the Dutch government every year issues a “zip code-SES” conversion table, which is used for civil and research purposes. This SES-proxy appears a fairly accurate individual SES proxy. The PRN is a national registry that contains validated and linked data from midwives, gynaecologists, general practitioners and paediatricians on 95% of all pregnancies with a minimal gestational age of 16 weeks. More information on these databases and the probabilistic linkage can be found in Supplement 1. All pregnancies with a delivery date between January 1999 and December 2014 were included. To be able to define drug dispensing in the 12-month period before conception and 12 months after delivery, women needed to be registered in the community pharmacy database of the PHARMO Database Network for both of these 12-month periods.

### Drugs of interest

All drugs are coded according to the Anatomical Therapeutic Chemical (ATC) classification system (WHO Collaborating Centre for Drug Statistics Methodology n.d.). ATC codes of SSRIs start with N06AB (further defined onto the fifth level) and include citalopram, escitalopram, fluoxetine, fluvoxamine, paroxetine and sertraline. The SSRI drug-dispensing data contained the following information per dispensing: timing of dispensing (before, during or after pregnancy, based on dispensing date), regimen and quantity dispensed. For all other drugs, information was available on second ATC level, with timing only (before, during or after pregnancy). Only outpatient drug data are reported. Note that inpatient drug provision is exceptional, limited to pregnant patients who for other reasons are hospitalised for a longer period.

### Analysis

Women were defined as user of a SSRI during one of the three phases (12 months leading up to pregnancy, during pregnancy and 12 months after pregnancy) if a dispensing was recorded of at least 28 days duration, for a given phase. For trend analysis, data was grouped into 2-year periods (based on year of delivery) and the chi-square test for trends was used.

From the available data on use and pregnancy duration/delivery, we defined the following seven mutually exclusive patterns: (1) women who used SSRIs before pregnancy only; (2) women who used SSRIs both before and during pregnancy; (3) women who used SSRIs before, during and after pregnancy; (4) women who used SSRIs during pregnancy only; (5) women who used SSRIs both during and after pregnancy; (6) women who used SSRIs after pregnancy only and (7) women who used SSRIs before and after pregnancy, but not during (recidivist). Percentages of each group were calculated per 2-year time period, allowing to test whether dispensing pattern changed, in particular related to the introduction of the guideline (2012).

To examine the association between general patient characteristics and continuation of SSRIs during pregnancy, only data of women using SSRIs in the year before pregnancy (women from the patterns 1, 2, 3 and 7) were used. Characteristics studied were year of delivery, parity, socio-economic status (based on data per zip code from the Central Bureau of Statistics (CBS), subdivided into low, middle and high), co-dispensing of psycholeptics (ATC code N05, including antipsychotics, anxiolytics, hypnotics and sedatives) and number of other co-medications (sum of all other pharmacy registered dispensed drugs, excluding SSRIs and psycholeptics) in the year before pregnancy. First, we determined univariable associations between the dependent variable and all the independent variables. Variables with a *p* value < 0.10 were entered into a multivariable logistic regression model. Independent variables with a two-sided *p* value < 0.05 in the multivariable model were defined as statistically significant. All associations were expressed as adjusted odds ratios (ORs) with the respective 95% confidence intervals (95% CIs). Statistical Package for Social Sciences (SPSS) version 25.0 was used.

## Results

A total of 153,952 pregnancies in the Netherlands with a delivery date between January 1999 and December 2014 were identified, with their associated dispensing data. Mean maternal age at delivery was 31 years, and socio-economic status was low in 28%, middle in 34% and high in 38%. Of these 153,952 pregnancies, 7284 (4.7%) used SSRIs in one or multiple phases. Mean maternal age at delivery for these pregnancies was similar (31 years), and socio-economic status differed slightly (shift to lower SES), with SES low in 35%, middle in 33% and high in 32% (*p* < 0.01). A short duration dispensing (< 28 days) was present in 536 (0.35% of all) women in the year before pregnancy, in 63 (0.04%) women during pregnancy and in 260 (0.17%) women in the year after pregnancy. These 759 cases were excluded from analysis.

### Dispensing patterns over time

Table 1 shows SSRI dispensing rates in the year before pregnancy, during pregnancy and in the year following pregnancy. Dispensing rate is highest in the year before pregnancy (e.g. 3.9% in 2013/2014) and lowest during pregnancy (e.g. 2.1% in 2013/2014). A significant rise over the years can be observed for all phases (before pregnancy, chi-square for trend = 48.411, *p* < 0.001; during pregnancy, chi-square for trend = 141.735, p < 0.001; after pregnancy, chi-square for trend = 10.540, *p* = 0.001), with the largest increase during pregnancy. The guideline introduction in 2012 did not interrupt this gradual increase.

**Table 1:** Number of deliveries in which the women received a dispensing for a selective serotonin reuptake inhibitor (SSRI) in the year before pregnancy, during pregnancy or in the year following pregnancy, per 2-year group based on delivery date

	Total number of deliveries	SSRI dispensing during:		
		The year before pregnancy (%)	Pregnancy (%)	The year following pregnancy (%)
1999/2000	6172	170 (2.8)	50 (0.8)	133 (2.2)
2001/2002	11,625	386 (3.3)	118 (1.0)	292 (2.5)
2003/2004	14,201	437 (3.1)	181 (1.3)	388 (2.7)
2005/2006	20,684	639 (3.1)	276 (1.3)	521 (2.5)
2007/2008	28,231	935 (3.3)	425 (1.5)	742 (2.6)
2009/2010	28,220	987 (3.5)	462 (1.6)	764 (2.7)
2011/2012	22,652	906 (4.0)	493 (2.2)	681 (3.0)
2013/2014	22,167	856 (3.9)	461 (2.1)	677 (3.1)

**Fig. 1:**
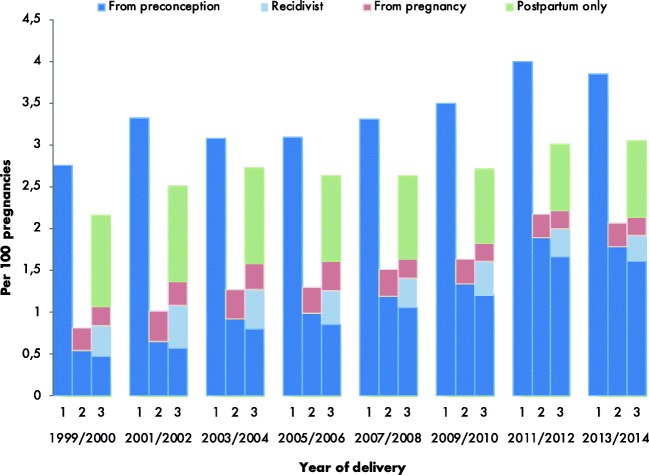
Selective serotonin reuptake inhibitors (SSRIs) perinatal dispensing rate per time period of interest per 100 pregnancies in the Netherlands, 1999–2014. 1 = the year before pregnancy, 2 = during pregnancy, 3 = the year after pregnancy. Complete data on all three phases was available for all women and thus the number of women at risk for each bar (1, 2 and 3) per 2 years is comparable. For example, a woman with a delivery date in 1999, who took SSRIs during all three phases (before, during, after) is represented in blue in all three bars (1, 2, 3) of the 1999 column. The dark blue bars represent the women that used their SSRI since the year before pregnancy. The pink and green bars represent the women that did not use SSRIs in the year before pregnancy but started using them during or after pregnancy respectively. The light blue bar represents those women that used SSRIs before pregnancy, discontinued during pregnancy, but restarted in the year after pregnancy

**Fig. 2:**
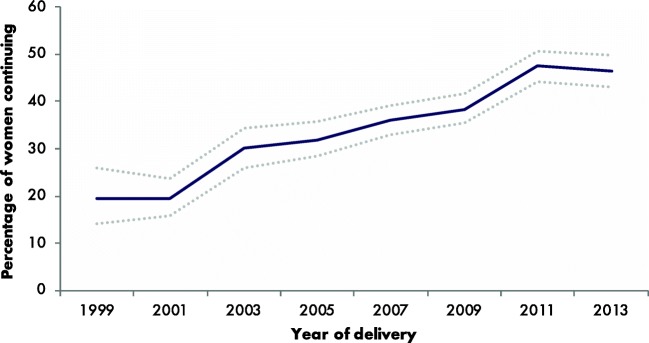
Percentage of women with selective serotonin reuptake inhibitor (SSRI) dispensing in the year before pregnancy continuing during pregnancy in the Netherlands, 1999–2014. Dotted lines represent the 95% confidence intervals

Figure 1 shows starting and stopping patterns of SSRI use, comparing 2-year groups. The figure visualises the seven groups as mentioned in the methods. In the early years of our cohort, a minority of women using SSRIs in the year before pregnancy continued SSRIs during pregnancy (19% in 1999/2000), while after 2012, this percentage increased substantially; 46% continued their medication (chi-square for trend = 25.256, *p* < 0.001) (Fig. 2). The percentage of women initiating medication during pregnancy remains constant (0.27% in 1999/2000 and 0.28% in 2013/2014). A small proportion (17.6% overall) of women who discontinued SSRIs during pregnancy restarts SSRIs postpartum (“recidivist,” pattern 7). Finally, the percentage of women who initiated SSRIs postpartum (pattern 6) decreased from 1.08% in 1999/2000 to 0.91% in 2013/2014. No obvious change in trend is visible after introduction of the guideline.

Concomitant use of psycholeptics was highly prevalent: Of the 5316 women using SSRIs in the year before pregnancy, 44.6% also used psycholeptics in that year. A mean number of 4.0 (SD 2.7) other co-medications were used in the year before pregnancy by this group (Table 2).

**Table 2:** Characteristics of all women with SSRI use (*n* = 7284) before, during and/or after pregnancy and of women with SSRI use (*n* = 5316) in the year before pregnancy

	Full SSRI sample	Women with SSRI use before pregnancy
Age in years, mean (SD)	31.5 (4.7)	31.5 (4.8)
Socio-economic status		
Low	2451 (33.6)	1713 (32.2)
Middle	2420 (33.2)	1816 (34.2)
High	2371 (32.6)	1759 (33.1)
Concomitant use of psycholeptics, yes (%)	2729 (37.5)	2369 (44.6)
Number of co-medications, mean (SD)	4.0 (2.7)	4.0 (2.7)

Table 3 shows, through a multivariable model, that several patient characteristics were independently associated with the decision to continue SSRI use. A low SES was related to decreased odds of continuation (OR 0.87, 95%CI 0.75–1.00) while a higher parity increased the odds of continuation with 15% per additional pregnancy (OR 1.15, 95%CI 1.09–1.21, *p* < 0.01). Specifically, the concomitant use of psycholeptics halved the probability of continuation of SSRI use during pregnancy (OR 0.50, 95%CI 0.43–0.55, *p* < 0.01). Generally, a more recent delivery increased the odds of continuation with 10% per calendar year (OR 1.10, 95%CI 1.08–1.11, *p* < 0.01);

**Table 3:** Univariable and multivariable associations between patient characteristics and continuing pre-conceptive selective serotonin reuptake inhibitor (SSRI) use during pregnancy. aOR = adjusted odds ratio, CI = confidence interval

	Univariable outcome		Multivariable outcome	
	cOR (95%CI)	*p* value	aOR (95%CI)	*p* value
Year of delivery	1.10 (1.08–1.12)	< 0.01	1.10 (1.08–1.11)	< 0.01
Parity	1.15 (1.09–1.22)	< 0.01	1.15 (1.09–1.21)	< 0.01
Socio-economic status				
Low	0.86 (0.75–0.98)	0.03	0.87 (0.75–1.00)	0.05
High	0.99 (0.87–1.13)	0.88	0.97 (0.84–1.11)	0.64
Concomitant use of psycholeptics	0.47 (0.42–0.53)	< 0.01	0.50 (0.43–0.55)	< 0.01
Number of co-medications	0.98 (0.96–1.00)	0.03	1.00 (0.98–1.02)	0.92

### Individual drugs

**Fig. 3:**
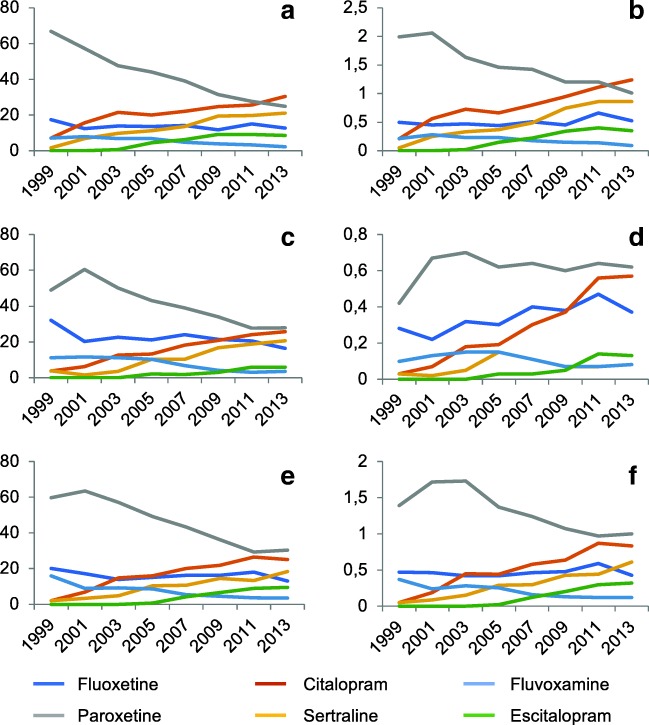
Relative and absolute dispensing rates per specific selective serotonin reuptake inhibitor (SSRI) before, during and after pregnancy in the Netherlands, 1999–2015. **a** Relative SSRI dispensing in the year before pregnancy. **b** Absolute SSRI dispensing in the year before pregnancy. **c** Relative SSRI dispensing during pregnancy. **d** Absolute SSRI dispensing during pregnancy. **e** Relative SSRI dispensing in the year after pregnancy. **f** Absolute SSRI dispensing in the year after pregnancy. Each year represents two calendar years

Of the six SSRIs dispensed, combining all years, paroxetine was the most frequently dispensed, accounting for 42.3% in the year before pregnancy, 41.3% during pregnancy and 46.0% in the year following pregnancy. However, a significant change was observed (Fig. 3). Over time, the absolute number of paroxetine dispenses decreased steeply (except during pregnancy), as did the share of this particular SSRI among users more generally. Reversely, citalopram and sertraline showed an increase over time. Still, paroxetine was the most often dispensed SSRI in 2013/2014.

Of the 1986 women who used SSRIs in the year before pregnancy (all years combined) and who continued medication during pregnancy, 127 women (6.4%) switched from SSRI at some point before, during or after pregnancy with no time trends being present (Table 4).

**Table 4:** Specific SSRI’s used before and after switch in women using SSRIs in the year before pregnancy and continuing SSRIs during pregnancy and switching at some point before or during pregnancy. Numbers sum up to more than the number of women due to multiple SSRIs used or multiple switches

	SSRI used before switch, *n* (%)	SSRI used after switch, *n* (%)
Citalopram	30 (22.2)	34 (21.1)
Escitalopram	10 (7.4)	9 (5.6)
Fluoxetine	11 (8.1)	27 (16.8)
Fluvoxamine	5 (3.7)	12 (7.5)
Paroxetine	31 (23.0)	41 (25.5)
Sertraline	48 (35.6)	38 (23.6)

## Discussion

This large 16-year population-based study shows a general increase of SSRI use throughout pregnancy and beyond. A substantial change of practice towards more continuation during pregnancy was present, initiated already before introduction of the 2012 guideline. The dominance of paroxetine as SSRI of choice decreased. The latter change is a change in general preference, as switching between specific SSRIs is rare (6.4%). Higher parity increased the continuation rate, while low SES and concomitant use of psycholeptics substantially decreased the continuation rate.

Two Dutch author groups previously reported on older and smaller samples. Ververs described the experience of a cohort of 29,005 deliveries between January 2000 and July 2003, which was made available from one healthcare insurance company (Ververs et al. 2006). Here, 2.2% of women used SSRIs before pregnancy, with a decrease to 1.4% in the third trimester and an increase to 2.3% in the post-delivery period. Bakker et al. reported on a cohort of 14,902 pregnancies with deliveries between 1995 and 2004 available from the Interaction Database (IADB.nl) (Bakker et al. 2008). Over these years, the exposure rate to SSRIs in the year preceding delivery increased from 1.2 to 2.9%. The data on the early years of our cohort fits to these observations and show that the growing trend has persisted 10 years beyond 2004. Research among the general Dutch population within the age category of 25 to 49 years (thereby including our target population) also reported an increase in SSRI use from around 2.5% in 1998 to 4.0% in 2004 (Bijlsma et al. 2014). A recent cohort study examined SSRI prescription in several European countries over the years 2004 to 2010. Prescription rates before, during and after pregnancy in our study were comparable to Denmark (4.1%, 2.3% and 4.1% respectively) and Italy (4.4%, 1.6% and 2.4% respectively), but prescription rates in the UK were considerably higher with 8.8 to 9.6% of women using SSRIs in the year before pregnancy (Charlton et al. 2015). Compared to the USA, where SSRI prescription rates during pregnancy increased from 2.9% in 1999 to 10.2% in 2003, prescription rates in European countries are low (Cooper et al. 2007).

A trend towards more continuation is observed over the years, without an obvious change in trend after introduction of the 2012 continuation guideline (NVOG 2012). As stated by this guideline, the recommendation to continue is a consequence of insufficient evidence on the benefits and risks to stop SSRI use, with or without non-pharmacological alternatives. Part of that is the absence of valid data on depression relapse after discontinuation. We interpret the low prescription rates in the Netherlands relative to other countries, such as the USA, as a higher prescription threshold, i.e., we assume that Dutch women with an SSRI prescription have a more severe psychiatric disorder. As this generally implies an increased risk of relapse, the continuation of SSRIs during pregnancy is even more justified. The alternative explanation is that severe psychiatric disorders, requiring pharmaceutical treatment, are more prevalent in other countries than in the Netherlands, although it is unlikely for this to fully explain the difference in SSRI prescription rates (Lim et al. 2018). We were surprised by the size of concomitant use of psycholeptics, as such drugs are universally associated with severe psychiatric disease. Their use halves the probability of continuation of SSRI use during pregnancy. The guideline does not address the issue. We hypothesise self-tapering by the women is responsible, as they may fear a cumulative detrimental effect on the unborn baby from multiple psychotropic medication; by self-tapering such women may think this benefits child outcome. Final interpretation of this interaction pattern requires more data, e.g. on the severity of underlying disease, length of medication use and previous discontinuation attempts and preferably interview data, both with the patients in question as well as associated caregivers guiding treatment decisions. A higher parity was associated with increased odds on continuing medication. This could be among others explained by the correlation of parity with age. A higher parity could point to a longer duration of psychiatric disease and/or longer duration of antidepressant treatment, which in turn might lead to an increased perceived dependence on antidepressant treatment (Singh et al. 2016). Another explanation could be that multiparous women with previous healthy infant(s) under SSRI use are more convinced of the safety of SSRI use during pregnancy and are therefore less likely to discontinue. Third, higher parity could mean a more stressful environment for the woman, thus contributing to the decision to continue medication.

There has been a major shift in specific SSRIs dispensed over the years, with paroxetine losing its popularity. This may be a reflection of evidence showing a higher rate of negative birth outcomes in paroxetine use compared to the other SSRIs (Alwan et al. 2007; Bakker et al. 2010; Berard et al. 2007; Cole et al. 2007; Louik et al. 2007; Nakhai-Pour et al. 2010; Wurst et al. 2010). Overall, paroxetine remained the most frequently dispensed SSRI, while a population-based study from Denmark, Iceland, Norway and Sweden showed paroxetine was the least prescribed SSRI in the period of 2008 to 2012 (Zoega et al. 2015).

Evidence on the risk of relapse of depression when discontinuing medication during pregnancy is insufficient. The first randomised controlled trial is being executed at the moment (Molenaar et al. 2016), but so far, only two naturalistic studies report on relapse rates in women continuing or discontinuing antidepressants during pregnancy (Cohen et al. 2006; Yonkers et al. 2011). Where the first study reported a significant increased risk of relapse in women who discontinued their medication compared to women continuing medication (86 vs. 26%), the second study did not find a significant influence of antidepressant discontinuation on relapse risk (16% overall). In our current study, only a small proportion of women restarted medication after discontinuation during pregnancy, which may reflect the relapse rate of the psychiatric disorder.

### Strengths and limitations

The size and composition of the Outpatient Pharmacy Database is a major strength of this study. It includes representative data on approximately 25% of the Dutch population, thereby allowing for fairly good estimates on the level of the Dutch population. Data was available from a year before conception until the end of the year following pregnancy. An accurate conception date could be obtained from the PRN database based on ultrasound or the last menstrual period, and the exact delivery date was also present in the PRN database. However, even this dataset had its limitations. Coverage of all pregnancies was less than 25% as linkage could not be established in all cases, potentially reducing representativeness. Exact timing of drug dispensing for this study was defined according to before, during and after pregnancy, enabling us to rule out the possibility that drugs dispensed just before pregnancy were still being used in the first trimester of pregnancy and so on. For some drug dispensing, an unknown ATC code was registered, thereby potentially missing a small amount of SSRI dispensing leading to underreporting of SSRI dispensing among the target population. Besides defining use in one of three phases by a prescription for a duration of 28 days, we did not take overall length of use into account.

A limitation of prescription registry data is that actual use may be less (non-compliance), or more (external sources of medication, shelf medication). Non-compliance is the most likely weakness, due to an increasing societal and professional reluctance to take/prescribe drugs during pregnancy, and psychotropic drugs in particular (Lupattelli et al. 2015). In this study, we assume the likelihood of underestimation to be very small. Last, information on pregnancies that ended before a gestational age of 16 weeks was excluded in our study as the PRN database only contains information of pregnancies of ≥ 16 weeks of gestation. However, SSRIs do not seem to increase risk of miscarriage (Andersen et al. 2014) and in addition, patterns examined in this study would not be affected.

## Conclusion

For more than a decade, perinatal SSRI use shows a steady increase. Rise is most prominent during pregnancy, by the combined effect of a general rise in SSRI use in all patients (possibly as a result of a rise in depression), and a change towards continuation rather than discontinuing when women get pregnant. Despite a substantial shift in drug preference, paroxetine is still most commonly used. Switches are rare. In view of the demonstrated impact of uncertainty regarding effectiveness and safety of SSRIs in pregnancy, future research should involve more detailed outcome research of SSRIs as it is and research into viable alternatives (drug/non-drug) for the use of SSRIs in pregnancy.

## Electronic supplementary material

ESM 1 (DOCX 15 kb)

## Footnotes

1 In this study defined as the entire period of pregnancy, the preconception period and the postnatal period (first year after birth).
