# Supplementary material for: Dispensing patterns of selective serotonin reuptake inhibitors before, during and after pregnancy: a 16-year population-based cohort study from the Netherlands
Source: Arch Womens Ment Health. 2019 Feb 14;23(1):71–9. doi: 10.1007/s00737-019-0951-5 (PMC6987060; doi:10.1007/s00737-019-0951-5)
Supplement: Supplementary file 1 — (DOCX 15 kb) [file 737_2019_951_MOESM1_ESM.docx]

**Supplement 1**

*Data sources*

The PHARMO Database Network is a dynamic cohort of participants that includes, among other information, drug-dispensing records from community pharmacies for more than three million individuals in the Netherlands (approximately 25% of the Dutch population) collected since 1998 (Herings et al. 1992). The PRN is a national registry that contains validated and linked data from four independent databases: the national obstetric database for midwives (LVR-1), the national obstetric database for gynaecologists (LVR-2), the national obstetric database for general practitioners (LVR-h) and the national neonatal/paediatric database (LNR) (Stichting Perinatale Registratie Nederland 2011). The registry contains information about care before, during and after delivery as well as maternal and neonatal characteristics (determinants) and outcomes of 95% of 175.000 pregnancies annually in the Netherlands, with a minimal gestational age of 16 weeks. Pregnancy duration is based on ultrasound or last menstrual period, as recorded in the PRN. The linkage method used here has been described elsewhere; it is generally based on the birth dates of the mother and the child and their approximate address (postal zip codes; covers 2000-5000 persons, 20-50 deliveries annually) (Houweling et al. 2013). The probabilistic record linkage here used techniques validated before with these datasets. Due to the favourable data characteristics in this case, accuracy is close to perfect (Méray et al. 2007; Tromp et al. 2005).

Herings, R.M., et al., *Pharmaco-morbidity linkage: a feasibility study comparing morbidity in two pharmacy based exposure cohorts.* J Epidemiol Community Health, 1992. **46**(2): p. 136-40.

Houweling, L.M., et al., *First year of life medication use and hospital admission rates: premature compared with term infants.* J Pediatr, 2013. **163**(1): p. 61-6 e1.

Méray, N., et al., *Probabilistic record linkage is a valid and transparant tool to combine databases without a patient identification number.* J Clin Epidemiol, 2007. **60**(9): p. 883-91.

Stichting Perinatale Registratie Nederland, 2011. *Grote Lijnen 10 Jaar Perinatale Registratie Nederland.* Utrecht.

Tromp, M., et al., *Medical Record Linkage of Anonymous Registries without Validated Sample Linkage of the Dutch Perinatal Registries.* Stud Health Technol Inform, 2005. **116**: p. 125-30.
